# Research on the use of tetracaine hydrochloride jelly surface anesthesia in combination with intravenous anesthesia for pain-free cystoscopy

**DOI:** 10.3389/fmed.2025.1653269

**Published:** 2025-09-03

**Authors:** Wenjie Bai, Daxun Luo, Mule Wurige, Bo Xiao, Jianxing Li

**Affiliations:** Department of Urology, Beijing Tsinghua Changgung Hospital, School of Clinical Medicine, Tsinghua University, Beijing, China

**Keywords:** tetracaine hydrochloride jelly, propofol, surface anesthesia, intravenous anesthesia, painless cystoscopy

## Abstract

**Objective:**

This study aimed to evaluate the efficacy and safety of tetracaine hydrochloride jelly surface anesthesia in combination with intravenous anesthesia for painless cystoscopy.

**Methods:**

This study was conducted at the Department of Urology, Beijing Tsinghua Changgung Hospital, from 1 January 2023 to 31 December 2024. A total of 60 eligible inpatients were recruited based on predefined inclusion and exclusion criteria and were randomly allocated to two groups, each comprising 30 patients. The experimental group received tetracaine hydrochloride jelly in conjunction with intravenous anesthesia, whereas the control group received intravenous anesthesia alone. Both groups were administered a slow intravenous infusion of 1.5 mg/kg propofol (1%) and 0.15 μg/kg remifentanil. After 2 min, the experimental group received 10 mL of tetracaine hydrochloride jelly via the urethra, while the control group received 10 mL of glycerin. The surgical procedure commenced 4 min later. Throughout the procedure, patients maintained spontaneous breathing, and additional propofol was administered if necessary. Preoperative parameters, including propofol dosage, cystoscopy time, anesthesia/awakening time, and relevant vital signs, were recorded for both groups.

**Results:**

Compared to the control group, the experimental group required significantly less additional propofol, had shorter anesthesia, cystoscopy, and surgical times, and exhibited higher postoperative SpO₂ levels. No postoperative complications or adverse events were observed in either group.

**Conclusion:**

The combination of tetracaine hydrochloride jelly surface anesthesia and intravenous anesthesia demonstrated significant advantages in painless cystoscopy, effectively reducing the additional dosage of propofol, shortening examination time, improving postoperative oxygenation, and ensuring high safety. This approach has considerable clinical value and potential for widespread application.

**Clinical trial registration:**

ChiCTR2300070527, Date of registration:2023-04-14.

## 1 Background

Cystoscopy (1) is a commonly used technique in urological endoscopic diagnosis and treatment. It is primarily utilized for the diagnosis and partial treatment of bladder and urethral diseases in clinical practice (2, 3), and is also an indispensable therapeutic modality in the management of urinary system stones. Traditionally, cystoscopy is performed under local anesthesia. However, pain and fear often hinder patient cooperation with physicians and may even lead to the termination of the procedure, posing significant challenges in clinical practice.

In recent years, with advancements in medical technology and the introduction of the concept of comfortable medical care, painless techniques have been widely applied in clinical settings. These techniques effectively address discomfort and complications caused by pain (4–6), greatly facilitating clinical diagnosis and treatment. However, in clinical practice, it has been observed that although intravenous general anesthetic agents provide significant analgesic and sedative effects, higher doses of anesthetic drugs are associated with increased anesthetic risks and reduced safety.

This study aims to explore whether the combination of tetracaine hydrochloride jelly for surface anesthesia and propofol for intravenous anesthesia can ensure effective analgesia and sedation while reducing the dosage of general anesthetics, thereby enhancing the safety of the procedure. The following results demonstrate the application value of tetracaine hydrochloride jelly surface anesthesia combined with intravenous anesthesia in painless cystoscopy.

## 2 Methods

### 2.1 General information

From 1 January 2023 to 31 December 2024, eligible patients admitted to the Department of Urology at Beijing Tsinghua Changgung Hospital were randomly assigned to two groups based on the inclusion criteria. The experimental group (*n* = 30) underwent painless cystoscopy with tetracaine hydrochloride jelly for surface anesthesia combined with intravenous anesthesia (Tetracaine + Intravenous Anesthesia Group), while the control group (*n* = 30) underwent painless cystoscopy with intravenous anesthesia alone (Glycerin + Intravenous Anesthesia Group). Preoperative parameters, including age, body mass index (BMI), gender, preoperative anesthesia classification (ASA grade), and preoperative vital signs, were recorded for both groups. This study adheres to the ethical principles of the 2024 version of the Declaration of Helsinki, ensuring that all research activities comply with the latest international standards of medical research ethics. This prospective randomized controlled trial was approved by the Medical Ethics Committee of Beijing Tsinghua Changgung Hospital (Ethical Approval Number: ChiCTR2300070527, Date of registration:2023-04-14). This study adheres to CONSORT guidelines.

### 2.2 Participation consent statement

This study was approved by the Ethics Committee of Beijing Tsinghua Changgung Hospital. All participants voluntarily signed informed consent based on a complete understanding of the purpose, process, potential risks, and benefits of the study. During the study, participants’ privacy and data security were strictly protected, and personal information was anonymized. Participants have the right to withdraw at any time or at any stage of the study without any adverse effects.

### 2.3 Inclusion and exclusion criteria

Inclusion criteria:

- Male patients aged ≥18 years who underwent urological stone surgery and had a unilateral ureteral stent placed postoperatively
- Good urethral condition
- Clear urine in the bladder
- No contraindications to general anesthesia
- Eligible for anesthesia assessment at the anesthesia clinic
- Provided informed consent to participate in the study

Exclusion criteria:

- Severe cardiopulmonary dysfunction, coagulopathy, or inability to tolerate general anesthesia
- Pregnant patients
- Patients considered unsuitable for painless cystoscopy by the anesthesiologist
- Patients with a urethral stricture that prevents cystoscopy
- Patients who did not sign the informed consent form 1.3 Surgical Procedure

Patients were required to fast for 8 h and abstain from drinking for 4 h before the procedure. Intravenous access was established, and continuous monitoring of blood pressure (BP), heart rate (HR), and oxygen saturation (SpO₂) was initiated at intervals of every 2 min. Nasal cannula oxygen was administered at a flow rate of 6 L/min, and routine preparations for intravenous anesthesia were completed.

In this study, all surgical procedures were performed by the same experienced urologist. Patients were placed in the lithotomy position. Routine disinfection and draping were performed. Before the start of the procedure, both groups received a slow intravenous injection of 1% propofol at a dose of 1.5 mg/kg and remifentanil at a dose of 0.15 μg/kg. After 2 min, the experimental group received 10 mL of tetracaine hydrochloride jelly via the urethra, while the control group received 10 mL of glycerin. Based on clinical experience, tetracaine hydrochloride jelly was found to provide the lowest pain VAS scores when cystoscopy was initiated 4 min after administration.

Therefore, surgical procedures were performed 4 min after urethral instillation in both groups. A 22 French rigid cystoscope was inserted via the urethra, and a foreign body forceps was used to remove the ureteral stent. The procedure was concluded upon withdrawal of the cystoscope. Patients maintained spontaneous breathing during the procedure, and an additional 1% propofol at 0.5 mg/kg was administered if signs of pain (e.g., frowning or limb movement) were observed.

### 2.4 Observation indicators

The primary observation indicator was the dosage of propofol used. Secondary indicators include cystoscopy time, anesthesia recovery time, and intraoperative and postoperative vital signs. The study assessed the correlation and significance of differences between these indicators.

### 2.5 Statistical methods

Data analysis was performed using SPSS 27.0 software. Categorical data were expressed as case numbers or percentages, and intergroup comparisons of categorical variables were conducted using the χ^2^ test. Normally distributed continuous variables were compared between groups using the independent t-test and expressed as mean ± standard deviation (x̄ ± s). Skewed continuous variables were compared using the Mann–Whitney U-test and expressed as the median (interquartile range) [50 (25, 75)]. A *p*-value of less than 0.05 was considered statistically significant.

## 3 Results

There were no statistically significant differences in baseline data between the two groups (Table 1). Compared with intravenous anesthesia alone, the combination of tetracaine hydrochloride jelly and intravenous anesthesia for painless cystoscopy resulted in lower additional doses of propofol (0 [0, 5] mL in the experimental group vs. 5 (5, 10) mL in the control group), shorter anesthesia time (5 [3, 70] min in the experimental group vs. 8 (4, 10) min in the control group), shorter cystoscopy time (5 [3, 9.75] min in the experimental group vs. 12 [9, 16] min in the control group), and shorter total surgical time (12 [9, 16.25] min in the experimental group vs. 22 [16, 26.5] min in the control group). Postoperative SpO₂ was higher in the experimental group (100 [99, 100]%) compared with the control group (99 [99, 100]%). Statistically significant differences were observed in the above indicators (*p* < 0.05). No statistically significant differences were found in systolic blood pressure, heart rate, and SpO₂ after induction, anesthesia recovery time, and systolic blood pressure, heart rate, and SpO₂ after the procedure between the two groups (*p* > 0.05). No complications or adverse events were observed in either group after the procedure (Tables 2, 3).

**Table 1:** Baseline data.

Parameters	Treatment group	Control group	*p* value
Patient count, number, %	30(50%)	30(50%)	
Age, year, mean ± SD	47.17 ± 11.219	50.90 ± 13.772	0.254
High, m, mean ± SD	172.77 ± 6.806	171.50 ± 5.871	0.443
Weight, kg, mean ± SD	75.17 ± 5.147	72.20 ± 7.425	0.078
BMI, kg/m2, mean ± SD	25.26 ± 2.26	24.53 ± 1.97	0.188
Preoperative systolic blood pressure, mmHg, mean ± SD	136.47 ± 16.67	134.27 ± 19.58	0.641
Preoperative respiratory rate, /min, 50[25,75]	16 [14,18]	15 [12.75,16]	0.119
Preoperative heart rate, /min, 50 [25, 75]	73.27 ± 8.436	73 ± 10.54	0.914
Preoperative SpO_2_, %, 50 [25, 75]	99 [98, 100]	99 [98,99]	0.219
Preoperative ASA Score, 50 [25, 75]	2 [2, 2]	2 [2, 2]	0.391

**Table 2:** Intraoperative data.

Parameters	Treatment group	Control group	*p* value
Anesthesia duration, min, 50 [25,75]	5 [3, 70]	8 [4, 10]	0.012
Endoscopy duration, min, 50 [25, 75]	5 [3, 9.75]	12 [9, 16]	p < 0.05
Total operation time, min, 50 [25,75]	12 [9, 16.25]	22 [16, 26.5]	p < 0.05
Post-induction systolic pressure, mmHg, mean ± SD	126.1 ± 14.194	127.27 ± 18.18	0.783
Post-induction heart rate, /min, mean ± SD	65.37 ± 7.31	67.93 ± 8.93	0.228
Post-induction SpO_2_, %, 50 [25, 75]	100 [99, 100]	99.5 [99, 100]	0.117
Intraoperative additional propofol dosage, ml, 50 [25, 75]	0 [0, 5]	5 [5, 10]	0.018

**Table 3:** Postoperative data.

Parameters	Treatment group	Control group	*p* value
Anesthesia recovery time, min, 50 [25, 75]	7 [4, 10]	7.5 [4.75, 10]	0.667
Post-exam systolic pressure, mmHg, 50 [25, 75]	125 [118, 140]	128 [109.75, 140.5]	0.609
Post-exam heart rate, /min, 50 [25, 75]	70 [64, 77.25]	70 [62, 76]	0.876
Post-exam SpO2, %, 50 [25, 75]	100 [99, 100]	99 [99, 100]	0.03
Complications, number	0	0	

## 4 Discussion

Cystoscopy is an essential diagnostic and therapeutic tool in urology. Initially, it was primarily performed under local anesthesia. However, with technological advancements, general anesthesia and painless techniques have been increasingly adopted to enhance patient comfort and procedural accuracy. While intravenous general anesthetic agents provide effective analgesia and sedation, cystoscopy remains an invasive procedure. In male patients, the physiological curvature of the urethra can cause friction between the cystoscope and the urethral mucosa or bladder wall during insertion, often leading to discomfort or pain. This may result in adverse reactions, such as mental tension, increased heart rate, and elevated blood pressure. These effects are particularly concerning in elderly patients with compromised cardiopulmonary function, as significant fluctuations in the respiratory and circulatory systems during the procedure can make patient cooperation difficult, increasing the risk of complications such as bleeding and perforation (7). Previous studies, such as those by Shahram et al. (8) have explored methods to alleviate pain during cystoscopy. For instance, injecting lidocaine into the glans penis was found to reduce pain during endoscope insertion.

In recent years, combined anesthesia techniques for cystoscopy have gained attention. Numerous studies have investigated the use of intravenous agents such as propofol in combination with flurbiprofen ester, tramadol, and fentanyl (9–12). However, the use of tetracaine hydrochloride paste for topical anesthesia in combination with intravenous anesthesia for painless cystoscopy has not been extensively studied. Tetracaine hydrochloride paste, a local anesthetic, works by blocking sodium channels, preventing the transmission of pain signals to the central nervous system. Based on this mechanism, our center conducted a prospective controlled study comparing tetracaine hydrochloride paste combined with intravenous anesthesia to intravenous anesthesia alone. The results demonstrated that the combined approach required less additional propofol, had shorter anesthesia, cystoscopy, and surgical times, and resulted in higher postoperative SpO₂ levels. These differences were statistically significant (*p* < 0.05).

Our findings align with those of Wang and Altementt (13, 14) who reported that combined anesthesia effectively reduces propofol usage without increasing cardiopulmonary complications during procedures. Although their studies focused on gastrointestinal endoscopy, the principles are similar, as both involve short-duration intravenous anesthesia for minor procedures. Thus, the conclusions drawn are largely in agreement with ours. In our study, we propose that tetracaine hydrochloride paste, as a local anesthetic, forms an anesthetic layer on the bladder mucosa, effectively mitigating the pain associated with cystoscope insertion and manipulation. This reduction in pain stimulus subsequently decreases the required dosage of general anesthetic agents, enhances procedural safety, and improves patient comfort. The experimental group exhibited a shorter anesthesia time compared to the control group. We attribute this to the local anesthetic effect, which enhances patient tolerance to cystoscope manipulation.

Consequently, interruptions or repeated positional adjustments due to pain or discomfort are minimized, leading to a smoother cystoscopy process and correspondingly shorter cystoscopy and surgical times. Similar observations have been made in studies where tetracaine was applied to gastrointestinal endoscopy, resulting in reduced procedural time, shorter physician manipulation time, and decreased patient discomfort. Regarding improved postoperative oxygenation in the experimental group, we speculate that this is due to the respiratory depressant effects of propofol (15). Since the combined anesthesia regimen reduces the dosage of propofol, its respiratory depressive effects are correspondingly diminished. As a result, patients can recover spontaneous breathing more rapidly after the procedure, thereby maintaining better oxygenation levels. Adequate oxygenation is essential for the normal functioning of various bodily systems postoperatively, facilitating accelerated metabolism, promoting wound healing, and reducing the incidence of postoperative complications.

This study has several limitations. As a single-center study with a relatively small sample size, it may be subject to selection bias and statistical randomness. Future research should expand the sample size and adopt a multicenter trial design to validate these findings. Additionally, future studies should compare the efficacy of tetracaine hydrochloride paste combined with intravenous anesthesia against other anesthetic regimens. Finally, this study did not assess patient or operator satisfaction and comfort levels, which are important indicators of anesthetic quality. These aspects should be included in future research to provide a more comprehensive evaluation.

## 5 Conclusion

The combination of topical tetracaine hydrochloride paste anesthesia and intravenous anesthesia offers significant advantages in painless cystoscopy. It effectively reduces propofol dosage, shortens procedure times, and improves postoperative oxygenation levels, all while maintaining high safety. This anesthetic approach holds considerable clinical value and warrants broader application and dissemination.

## Data Availability

The original contributions presented in the study are included in the article/supplementary material, further inquiries can be directed to the corresponding authors.
